# External Beam Therapy in a Four-Field Box Technique with Paclitaxel versus a Two-Field Technique with Cisplatin in Locally Advanced Carcinoma Cervix: A Phase II Monocentric Trial

**DOI:** 10.5402/2012/390193

**Published:** 2012-12-31

**Authors:** Vijayakumar Narayanan, Bibek Bista, Samir Sharma

**Affiliations:** ^1^Cancer Care Kenya, P.O. Box 39173, Nairobi, Kenya; ^2^B. P. Koirala Memorial Cancer Hospital, Bharatpur, Chitwan, Nepal

## Abstract

*Introduction*. External beam pelvic radiotherapy with cisplatin and brachytherapy is the standard of care for patients with advanced cervical malignancy. This study was aimed at evaluating the toxicity of a two-field radiotherapy with cisplatin and brachytherapy compared to a four-field box technique with paclitaxel and brachytherapy for stages IIB/IIIB cervical cancer. The differences in response to the overall treatment were also examined. *Methods*. 35 patients were enrolled in this phase II prospective randomized trial conducted from February 2006 to February 2007. In arm I, up to 40 Gy in 20 fractions followed by 10 Gy in 5 fractions in split field with cisplatin 40 mg/M^2^ and, in arm II, 50 Gy in 25 fractions with paclitaxel 50 mg/M^2^ were given. *Results*. Toxicity in genitourinary, lower gastrointestinal, and hematological tissues was significantly higher in arm I. The duration of concurrent chemoradiotherapy in either arm was similar. The overall treatment time was less in arm II. No statistically significant difference in the objective response was observed between arms. *Conclusion*. Two-field radiotherapy with cisplatin is a tolerable regime but more toxic than four-field box radiotherapy with paclitaxel. The major setbacks are that a radiotherapy technique as well as chemotherapy is different; hence, toxicity and outcome of treatment should be viewed as a collective response of the whole treatment regimen and the small sample size.

## 1. Introduction

The commonest gynecological cancer before the age of 50 is carcinoma cervix. The incidence is high in developing and underdeveloped countries. Women belonging to low socioeconomic status show higher incidence [1]. Though there is no population-based cancer registry in Nepal, incidence of the age-standardized incidence rate and mortality rate is estimated to be 26.4 per 100000 women and 14.1 per 100000 women, respectively. Similarly, the incidence of cervical cancer per 100000 women in India, Bangladesh, and Sri Lanka is estimated to be 30.7, 27.6, and 17.7, respectively [2]. Poor personal hygiene, poor nutritional status, multiple sexual partners, first coitus in young age, early child birth, promiscuity of the spouse, human papilloma virus infection, sexually transmitted diseases, and immunocompromised states are cited as main risk factors [3, 4]. Though there is tremendous breakthrough in cancer research and changes in clinical practice, the nature of disease still remains the same.

Early carcinoma of the uterine cervix can be effectively managed either by surgery or by radiotherapy; the results are equivocal [5]. A randomized control trial (RCT) in Taiwan evaluating the side effects and quality of life of surgery versus radiotherapy in early cervical cancer showed that though initial complications were different, long-term complication and quality of life were similar in both modalities [6]. Patients with stages IIB and III are treated with irradiation alone. Concomitant use of chemotherapeutic agents as radiosensitizer has shown to be beneficial in several randomized trials [7–9]. All positive trials showed a 43–46% reduction in the risk of recurrence and death, translating into 16% absolute benefit in disease-free survival and 12% absolute benefit in total survival. The trials also showed a reduction in the rate of distant metastasis in the chemoradiotherapy arm. Two meta-analyses confirmed the finding [10, 11]. The National Comprehensive Cancer Network Guidelines [12] categorically recommend pelvic radiotherapy with concurrent cisplatin containing chemotherapy (Category I) and brachytherapy for advanced cervical cancer. A recent meta-analysis involving 18 RCT showed that chemoradiotherapy has better 3- and 5-year survival rate compared to radiotherapy alone while the adverse effects were not statistically different [13].

Thus, chemoradiotherapy has been the standard rather than an option for treatment of advanced cervical cancer. However, there lies the problem of toxicity, and in order to circumvent this, trials are on the way to evaluate the new drugs and different dosing schedules, which may result in a more acceptable toxicity profile. A prospective multicentric study in eight Asian countries showed that concurrent chemotherapy using cisplatin is feasible and produces good survival outcome and reduced adverse effects [14].

Radiation therapy in cervical cancer has made significant advances in the past few decades. It is now suggested that a Point A dose of 85–90 Gy is the optimum in cervical cancer [15]. This dose is achieved by external beam radiotherapy and brachytherapy. Despite all these advances, treatment response in advanced cancer of the cervix has a plateau of 30–45% at 5 years. The situation is still worse in developing and underdeveloped countries, where the data available is often heterogenous. Poor randomization, inadequate sample size, nonuniform usage of chemotherapeutic drugs, poor documentation, and irregular followup are pointed out as fallacies of the trials. Follow-up investigations are different between a developed and an underdeveloped country. Investigations by magnetic resonance imaging (MRI) and positron emission tomography (PET) scans are routinely performed in the former while in the latter they are neither easily accessible nor affordable for routine use. In this phase II randomized study conducted in our institute, we compared one group of patients treated with external beam radiation in a two-field technique with weekly cisplatin as per the institutional protocol to a second group treated with a four-field box technique with weekly paclitaxel. The primary aim of the study was the evaluation of toxicity of the two regimes in an underdeveloped country setting. Clinical management of cancer patients in an underdeveloped country has its own challenge such as absence of infrastructure, nonavailability of specialist services, affordability of treatment cost, and accessibility of care. So, the result of this study can be a clinical guide to a radiation oncologist in such setup.

The institutional ethics committee approved the study as per the institutional rules.

## 2. Patients and Methods

### 2.1. Patients

The study enrolled patients with a pathologically confirmed carcinoma cervix FIGO stages IIB and IIIB. Eligibility criteria included an Eastern Cooperative Oncology Group (ECOG) performance status >2, chemotherapy naive, prior treatment score 0 status, and negative para-aortic nodes.

Grading of toxicity in lower gastrointestinal, genitourinary, and hematological tissues was done as per the Radiation Therapy Oncology Group/European Organization for Research and Treatment of Cancer (RTOG/EORTC) [16] toxicity criteria. The gynecologist and radiation oncologist jointly evaluated the patients. Pretreatment evaluation included a complete hemogram, biochemical profile, X-ray chest, ultrasonogram of abdomen and pelvis, and cystoscopy.

### 2.2. Treatment Plan

This was a double-blind prospective randomized controlled study. So, a two-parallel-arm of treatment modality was developed. The patients were randomly allocated to treatment arms based on a computer generated random number.

### 2.3. External Beam Radiotherapy

Homogenous irradiation of the volume of tissue was planned in arm I by two parallel opposed anterior and posterior fields, in which the upper limit was on the lower border of L4 and the lower limit was at a 2-3 cm safety allowance from the lower extension of growth, usually at the inferior margin of obturator foramen. Lateral margins were kept 2 cm beyond a true pelvic brim. In arm II, the plan was a four-field box/brick, in which, apart from the anterior and posterior fields, two lateral fields were also set up. The anterior border of the target volume in the lateral portal was 4 cm anterior to the anterior margin of L5 vertebral body while the posterior border was 2 cm anterior to the sacral hollow, usually at S2/S3 junction. Treatment was given 5 days a week, 200 cGy/day, with all fields treated daily. In arm I, after a dose of 40 Gy, a midline block was placed and additional 10 Gy was given, whereas, in arm II, no split field was used; all patients were given 50 Gy. Toxicity assessments were made every week and in between, when found necessary.

### 2.4. Chemotherapy

In arm I, weekly cisplatin at a dose of 40 mg/M^2^ was given. Cisplatin is considered as the most cytotoxic drug for patients with advanced and recurrent squamous cell carcinoma cervix [17]. Cisplatin is thought to enhance cell death through cytotoxic DNA crosslinks, hypoxic cell sensitization, and inhibition of cell damage repair [18].

In arm II, patients were given weekly paclitaxel in a dose of 50 mg/M^2^. Paclitaxel is found to be effective and a well-tolerated radiosensitizer for patients with cervical cancer [19, 20]. In vitro studies on paclitaxel revealed potentiation of antitumor activity and recruitment of cells into most radiosensitive phases of cell cycle, the G2/M [21].

### 2.5. Brachytherapy

After completion of the external beam therapy, all patients were subjected to high-dose rate brachytherapy (HDBT), which was based on a Manchester triple source system comprising an intrauterine device and two vaginal ovoids. 21 Gy to Point A was given in three sessions, each at an interval of 1 week.

### 2.6. Parametrial Boost

In the patients in arm II in whom a significant distal parametrial disease was felt at the time of brachytherapy, an additional parametrial dose was given using opposed anterior and posterior fields with a half-beam block. 6–10 Gy in 3–5 fractions in 1 week, depending on the dose already given by external beam therapy and brachytherapy, was administered to boost the dose to a lateral parametrium to 60 Gy.

### 2.7. Definition of Response

Response to treatment was assessed in all patients included in the trial. Objective response was evaluated after 1 month after chemoradiotherapy as per the WHO criteria [22, 23]. Confirmation of the response was performed after 2 months. Complete response was defined as the disappearance of all disease; partial response was defined as at least a 30% decrease in the sum of the longest diameter (LD) of target lesion. Progressive disease was defined as at least 20% increase in the LD of target lesion. It was decided to evaluate the response earlier if there was any clinically evident or suspected progression of the disease.

### 2.8. Toxicity Criteria

Toxicity was evaluated in all patients who enrolled in treatment. Toxicity was graded according to the RTOG/EORTC criteria. Dose and schedule modifications were based on weekly blood counts and biweekly assessment of clinical toxicity. It was decided to interrupt the treatment in the event of Grade 2 or the previously mentioned hematological or Grade 3 or the previously mentioned nonhematological toxicity till resolution of the problem. It was also decided to withdraw patients from the trial in the event of any Grade IV toxicity.

### 2.9. Statistical Methods

*t*-test was used to compare the time required for completion of external beam therapy and for evaluation of the response to treatment, assuming that the variance was different in each group. Chi square test was used to evaluate the difference between the rate of severe complications (>G0) between the groups.

## 3. Results

From February 2006 to February 2007, the study was completed with 35 patients. In arm I, there were 16 patients and in arm II 19 patients. Patient characteristics are shown in Table 1.

### 3.1. Administration of Therapy and Toxicity

The therapy was administered in two phases. External beam radiotherapy and weekly chemotherapy, that is, the concurrent chemoradiotherapy (CCRT), were given in first phase and high-dose rate brachytherapy (HDBT) in the second phase.

The mean number of days required for completion of the first phase of the treatment, that is, CCRT in both groups, was 36 and 35 days, respectively. Assuming that the variance was different in each group, *t*-test was used. The value of *t* was calculated to be 0.67 and with degree of freedom of 36.05 (which was calculated assuming that the variance in both groups is not the same and unknown), and the *P* value >0.05, which is statistically insignificant. This indicates that time taken for treatment in arm II is statistically similar to that of arm I. The results are provided in Table 2. Difference in the rate of complication (>G0) as per RTOG/EORTC in both groups in the case of genitourinary system (GUS), lower gastrointestinal system (LGI), and hematological system was evaluated. The data is presented in Tables 3, 4, 5, 6, and 7 with the results.

## 4. Discussion

The primary end point of this study was toxicity related to concurrent chemo-irradiation. Hence, only patients with Eastern Cooperative Oncology Group (ECOG) performance status ≤2 were recruited for the trial. All of them were eligible for CCRT with either cisplatin or paclitaxel. A high dropout was expected in patients with a performance status more than 2, so they were excluded. All patients completed the first phase of their treatment, that is, CCRT, though statistically significant difference were observed in the study arms, in terms of genitourinary, lower gastrointestinal, and hematological toxicity. Paclitaxel plus the box technique was found to be less toxic than cisplatin plus a two-field technique (*P* < 0.001). It should be noted that the toxicity reported was self-limiting, requiring no interruption of treatment or no dose reduction. There is no significant difference in the duration of completion of external beam radiotherapy (XRT) with weekly chemotherapy in either arm.

This study has a major drawback. In this study, the technique of radiotherapy as well as the sensitizer chemotherapy is different in both arms. Hence, toxicity and the outcome of treatment should be viewed as a collective response of the whole treatment regimen. It is not possible to conclude that the statistically significant supremacy of one regimen over the other in terms of toxicity as an attribute of either the technique of radiotherapy or the radiosensitizer is used. Both factors had contributed to the less toxicity observed in arm II.

Only controlled trials can provide an answer to the superiority of one drug over other, but considering the economic aspect of both drugs in the context of a country where majority of the people are below poverty line, we would like to suggest Cisplatin as a radiosensitizer in concurrent chemo irradiation in advanced cervical cancer. Paclitaxel should be given to those patients who can afford the drug for minimizing toxicity and can be used in otherwise unfit patients, for example, those with impaired renal functions. Acceptable geometry for the conventional Manchester triple system placement following optimal tumor shrinkage was observed in arm II, where the treatment technique was four-field box. Such a response was not present in the two-field technique. In the box technique irradiation where two lateral portals are also used, there is dual advantage of the sparing portion of small bowel and bladder anteriorly and rectum posteriorly as well as yielding more dose to the tumor. In the two-field technique, the use of midline shield for splitting the portal after 40 Gy in order to spare the bladder and the rectum results in less dose to the tumor. Such a setting is not required in the box technique.

In the present study, we did not give any priority to the subjects and they were given the time slots for brachytherapy as per the existing waiting list. Being the only center in the entire country with HDBT facility, the waiting list is long. Thus, extrinsic factors also play a crucial role in the completion of treatment. Locoregional control is inversely proportional to the total duration of radiation treatment in carcinoma cervix [24, 25]. Even then, no statistically significant difference was observed in the response to treatment in either arm. The small sample (*n* = 35) is not adequate to find out the statistical significance in response rates. Large trials are necessary to arrive at a definite conclusion. The overall time to complete the treatment is less in arm II, partly due to less toxicity during treatment. In general, patients take more than 2 months for completion of the entire treatment, which is not desirable as it may adversely affect the outcome of treatment. The setback of radiotherapy services in Nepal is due to the lack of strategic planning. Increasing workload and inadequate resources potentially exacerbate inequalities in the standard of care. Waiting time for treatment is an inevitable consequence of inadequate resource, underutilization of the available resource, or both.

Considering the evidence-based oncology in cervical cancer, we find that the enormous amount of data available is inconvenient and is not appropriate in the context of a country like Nepal. We feel that our ultimate responsibility is to the individual patient, and to a management according to her predicament should be the prime concern.

## 5. Conclusions

CCRT is the standard of care in advanced cervical cancer. Two-field external beam radiotherapy with weekly cisplatin is a tolerable regime, but more toxic than the four-field box external beam radiotherapy with weekly paclitaxel. The treatment can be given in an outpatient setting and is easy to administer. Overall treatment time is significantly prolonged in arm I but no statistically significant difference was observed in terms of complete response at the third month of followup. Efforts should be made to minimize the period from initiation of treatment to completion. The treatment schedule should be flexible enough, to adapt the response of the tumor and normal tissue reactions, at the same time, minimizing the duration. 

## Figures and Tables

**Table 1:** Patient characteristics.

Characteristics	Arm I	Arm II
Number of patients	16	19
Age in years		
Median	42	50
Range	35–65	35–65
Histology		
Squamous cell carcinoma	16	16
Adenocarcinoma	0	3
Stage		
IIB	8	11
IIIB	8	8

**Table 2:** Treatment days for the first phase (CCRT).

Number of days taken to complete the first phase (CCRT)	Arm I	Arm II
Mean	36	35
Median	37	34
Range	30–41	32–42
SD	3.04	2.96

**Table 3:** Complications of the genitourinary system.

Genitourinary toxicity	Arm I	Arm II
Number of patients with G0 toxicity	8 (50%)	19 (100)
Number of patients with >G0 toxicity	8 (50%)	0 (0%)

Chi square test yielded a *P* value <0.001, which indicates that the proportion of patients developing genitourinary complications is significantly less in arm II.

**Table 4:** Complications of the lower gastrointestinal system.

Lower gastrointestinal toxicity	Arm I	Arm II
Number of patients with G0 toxicity	7 (46%)	12 (63%)
Number of patients with >G0 toxicity	9 (54%)	7 (37%)

Chi square value yielded a *P* value 0.016 (<0.05) which indicates that the proportion of patients developing the lower gastrointestinal complication is significantly less in arm II.

**Table 5:** Hematological complications.

Hematological toxicity	Arm I	Arm II
Number of patients with G0 toxicity	8 (50%)	19 (100%)
Number of patients with >G0 toxicity	8 (50%)	0 (0%)

Chi square test yielded a *P* value <0.001, which indicates that the proportion of patients getting hematological complications is significantly less in arm II.

**Table 6:** No. of days required to complete treatment.

Total number of days to complete the treatment	Arm I	Arm II
Mean	71.44	66.16
Median	69	64
Range	61–87	55–84
SD	7.74	7.88

Assuming that the variance was different in each group, a *t*-test was used. The value of *t* was calculated to be 1.99 and with a degree of freedom of 32.2 (which was calculated assuming that the variance in both groups is not the same and unknown); the *P* value <0.025, which is statistically significant. This indicates that time taken for overall treatment in arm II is significantly less than that of arm I.

**Table 7:** Response to treatment.

Number of patients with complete response/recurrence	Arm I	Arm II
CR after 3 followups	12 (75%)	18 (94.7%)
Recurrence	4 (25%)	1 (5.3%)
Complications		
Proctitis	1	0
Vulval edema	1	0
Pulmonary metastasis	1	0

Pulmonary metastasis, vulval edema, and proctitis were noted in one patient each in arm I. For comparison of differences in terms of response determined as per WHO, first two rows were used, and calculation yielded a *P* value 0.096 (Fishers exact test 0.156), which is statistically not significant.
